# The beneficial therapeutic effects of plant‐derived natural products for the treatment of sarcopenia

**DOI:** 10.1002/jcsm.13057

**Published:** 2022-08-12

**Authors:** Mohammad Bagherniya, Atena Mahdavi, Nafiseh Shokri‐Mashhadi, Maciej Banach, Stephan Von Haehling, Thomas P. Johnston, Amirhossein Sahebkar

**Affiliations:** ^1^ Department of Community Nutrition, School of Nutrition and Food Science Isfahan University of Medical Sciences Isfahan Iran; ^2^ Food Security Research Center Isfahan University of Medical Sciences Isfahan Iran; ^3^ Anesthesia and Critical Care Research Center Isfahan University of Medical Sciences Isfahan Iran; ^4^ Department of Clinical Nutrition, School of Nutrition and Food Science, Food Security Research Center Isfahan University of Medical Sciences Isfahan Iran; ^5^ Department of Hypertension, WAM University Hospital in Lodz Medical University of Lodz Lodz Poland; ^6^ Cardiovascular Research Centre University of Zielona‐Gora Zielona‐Gora Poland; ^7^ Department of Cardiology and Pneumology University of Göttingen Medical Center Göttingen Germany; ^8^ German Center for Cardiovascular Research (DZHK), partner site Göttingen Göttingen Germany; ^9^ Division of Pharmacology and Pharmaceutical Sciences, School of Pharmacy University of Missouri‐Kansas City Kansas City MO USA; ^10^ Biotechnology Research Center, Pharmaceutical Technology Institute Mashhad University of Medical Sciences Mashhad Iran; ^11^ Applied Biomedical Research Center Mashhad University of Medical Sciences Mashhad Iran; ^12^ School of Medicine The University of Western Australia Perth Australia

**Keywords:** Nutraceuticals, Medicinal plants, Sarcopenia, Inflammation, Muscle disorder

## Abstract

Sarcopenia is an age‐related muscle disorder typically associated with a poor quality of life. Its definition has evolved over time, and several underlying causes of sarcopenia in the elderly have been proposed. However, the exact mechanisms involved in sarcopenia, as well as effective treatments for this condition, are not fully understood. The purpose of this article was to conduct a comprehensive review of previous evidence regarding the definition, diagnosis, risk factors, and efficacy of plant‐derived natural products for sarcopenia. The methodological approach for the current narrative review was performed using PubMed, Scopus, and Web of Science databases, as well as Google Scholar (up to March 2021) in order to satisfy our objectives. The substantial beneficial effects along with the safety of some plant‐derived natural products including curcumin, resveratrol, catechin, soy protein, and ginseng on sarcopenia are reported in this review. Based on clinical studies, nutraceuticals and functional foods may have beneficial effects on physical performance, including handgrip and knee‐extension strength, weight‐lifting capacity, time or distance travelled before feeling fatigued, mitochondrial function, muscle fatigue, mean muscle fibre area, and total number of myonuclei. In preclinical studies, supplementation with herbs and natural bioactive compounds resulted in beneficial effects including increased plantaris mass, skeletal muscle mass and strength production, increased expression of anabolic factors myogenin, Myf5 and MyoD, enhanced mitochondrial capacity, and inhibition of muscle atrophy and sarcopenia. We found that several risk factors such as nutritional status, physical inactivity, inflammation, oxidative stress, endocrine system dysfunction, insulin resistance, history of chronic disease, mental health, and genetic factors are linked or associated with sarcopenia. The substantial beneficial effects of some nutraceuticals and functional foods on sarcopenia, including curcumin, resveratrol, catechin, soy protein, and ginseng, without any significant side effects, are reported in this review. Plant‐derived natural products might have a beneficial effect on various components of sarcopenia. Nevertheless, due to limited human trials, the clinical benefits of plant‐derived natural products remain inconclusive. It is suggested that comprehensive longitudinal clinical studies to better understand risk factors over time, as well as identifying a treatment strategy for sarcopenia that is based on its pathophysiology, be undertaken in future investigations.

## Introduction

The global elderly community is rapidly growing.^1^ Consequently, there will be a significant increase in age‐related disorders, for instance, sarcopenia and frailty.^2^ Sarcopenia is distinct as a muscle disorder and is associated with a poor quality of life, a risk of falling with potential bone fractures, and greater healthcare costs.^3^

**Figure 1:**
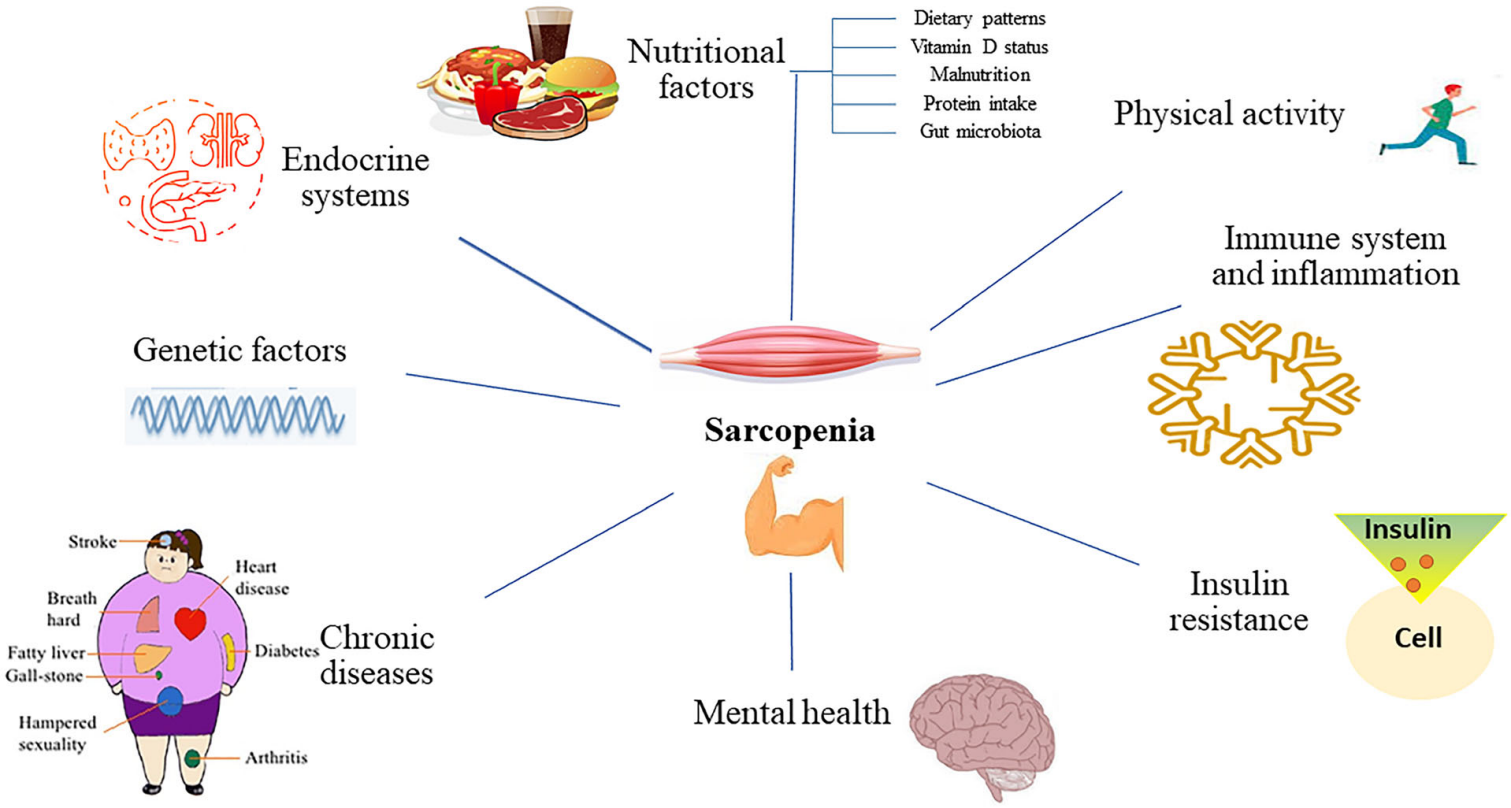
Risk factors associated with sarcopenia.

The pathophysiology of sarcopenia is multifactorial. Several underlying risk factors have been linked to the prevalence and development of sarcopenia, although they have not been fully elucidated. According to previous studies, nutritional status, physical activity, inflammation, genetic characteristics, endocrine function, and metabolic risk factors appear to play important roles in the development of sarcopenia. Figure 1 summarizes the primary risk factors *associated* with sarcopenia.

Due to the fact that there are no specific therapeutic options for sarcopenia, suitable nutrition, in combination with dietary supplements and regular exercise, is considered a practical strategy to delay the progression of sarcopenia.^4^ In recent years, nutraceuticals and herbal medicines have attracted significant attention for the prevention and treatment of sarcopenia.^5^, ^6^ These natural products are inexpensive, accessible, and safe (low toxicity) and could be used as a novel approach to help alleviate some of the underlying symptoms of sarcopenia. It is well documented that oxidative stress and inflammation have an important role in the pathogenesis of muscle damage and pain, sarcopenia, frailty, and several age‐related conditions including neurodegenerative diseases, CVDs, chronic kidney disease, COPD, and cancer.^7^, ^8^ It would appear reasonable that reducing oxidative stress and subsequent inflammation should have a significant impact on the prevention and/or treatment of muscle damage and sarcopenia. Fortunately, medicinal plants and natural herbal bioactive compounds are well‐known for their antioxidant, anti‐inflammatory, and anti‐aging properties.^9^, ^10^, ^11^, ^12^

The primary reason for the beneficial effects of these botanicals, as it pertains to the reduction of muscle inflammation, is that they modulate age‐related transcriptional factors in key biochemical pathways.^5^ Other beneficial effects of these botanicals on muscle involve the prevention of muscle damage, prevention of muscle atrophy, muscle regeneration and differentiation, muscle strengthening, and muscle resistance to fatigue during vigorous exercise.^5^ In almost all of the aforementioned muscle processes, antioxidant and anti‐inflammatory properties of medicinal herbs are involved.^5^ Below we summarized the results of previous preclinical and human studies conducted on sarcopenic patients using medicinal plants in an attempt to improve muscle function.

## Methods

**Figure 2:**
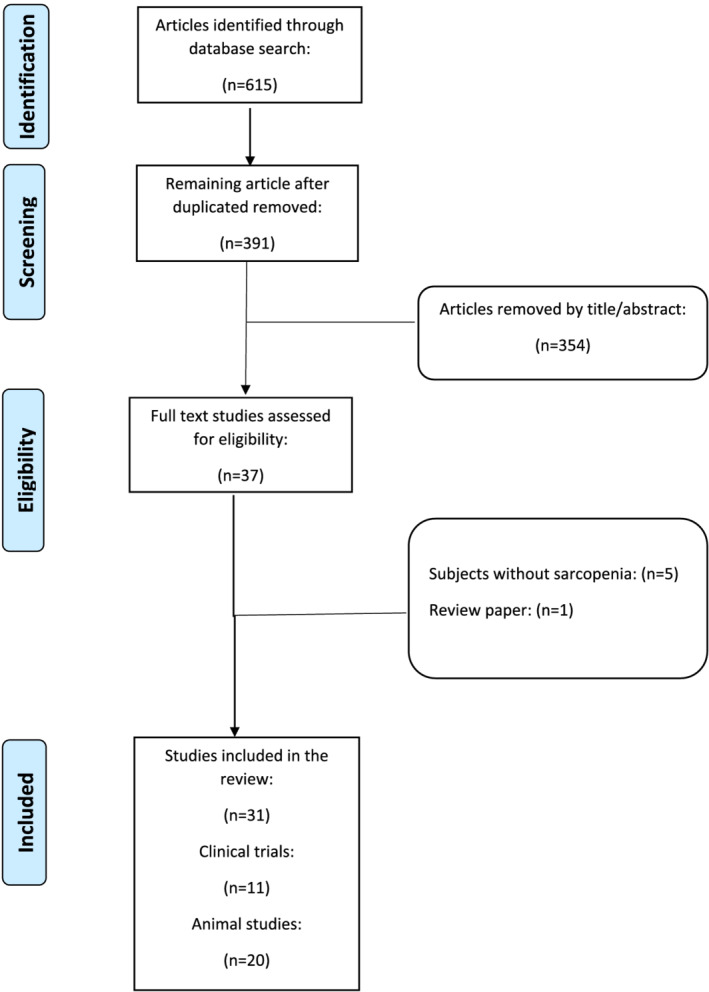
Flow chart of the process used for selection of the included studies.

The methodological approach for the current narrative review was performed using PubMed, Scopus, and Web of Science databases, as well as Google Scholar from inception up to March 2021 in order to accomplish our objectives. The comprehensive search was performed using keywords related to the definition, diagnosis, risk factors, and efficacy of plant‐derived natural products for sarcopenia without language restrictions to identify studies assessing the effects of herbal medicine on sarcopenia. The following keywords were used: (‘curcumin’ OR ‘curcuminoids’ OR ‘ *Curcuma Longa* ’ OR ‘turmeric’ OR ‘flaxseed’ OR ‘Cinnamon’ OR ‘Ginseng’ OR ‘Ginger’ OR ‘Green tea’ OR ‘Catechins’ OR ‘epicatechins’ OR ‘Sulforaphane’ OR ‘Silymarin’ OR ‘Silybin’ OR ‘black tea’ OR ‘Tea’ OR ‘Bayberry’ OR ‘Resveratrol’ OR ‘Anthocyanin’ OR ‘Garlic’ OR ‘Allicin’ OR ‘Quercetin’ OR ‘Berberine’ OR ‘Propolis’ OR ‘lycopene’ OR ‘Pomegranate’ OR ‘crocin’ OR ‘saffron’ OR ‘Flavonoid’ OR ‘Flavonoids’ OR ‘soy’ OR ‘isoflavones’ OR ‘natural herbal medicine’ OR ‘medicinal plants’ OR ‘medicinal plant’ OR ‘herbal’ OR ‘herb’ OR ‘Natural product’ OR ‘phytochemical’ OR ‘phytochemicals’) AND (‘sarcopenia’). Whenever possible, Medical Subject Headings (MESH) terms were used. The research procedures were performed by two investigators (M. B. and N. S.) separately and in duplicate. Any conflicting viewpoints/perspectives in this regard were decided *via* discussion with the third author (A. S.). In this study, all trials, which examined the effects of plant‐derived natural products and herbal medicine (use alone or in combination form) on sarcopenia, were included. There was no limitation with respect to age, and the number of underlying diseases of the participants included in the studies. In addition, all of the experimental studies, which assessed the effects of plant‐derived natural products (use alone or in combination form) on sarcopenic animals were included. Studies were excluded if they were (i) duplicate records; (ii) cellular and molecular studies; (iii) considered any other diseases except sarcopenia; and (iv) reviews, letters, editorial articles, or case reports. The search yielded a total of 615 references: ISI (*n* = 206), PubMed (*n* = 143), and Scopus (*n* = 266). After removing duplicate references, 391 records remained. The first 100 records of Google Scholar were also searched. Subsequently, the title and abstracts of the unduplicated studies were reviewed, and 360 irrelevant records were omitted. Finally, 31 references (clinical trials, *n* = 11, comprising 724 individuals and animal studies, *n* = 20) were included in the current narrative review (Figure 2).

### Curcumin

Curcumin, also called diferuloylmethane, is a principal polyphenol of turmeric, which is responsible for turmeric's yellow colour.^13^ In addition to the use of turmeric as a food spice, it is used traditionally as an herbal medicine in Asian countries.^14^, ^15^ Recently, it has been demonstrated that most of the health‐related advantages of turmeric are attributed to curcumin, because it has been reported that curcumin exhibits anti‐tumour, antioxidant, anti‐inflammatory, antithrombotic, chemopreventive, antimutagenic, anticancer, anti‐atherosclerotic (cardioprotective), lipid‐modifying properties, anti‐diabetic, antimicrobial, analgesic, pulmonoprotective, antidepressant, and anti‐aging properties.^16^, ^17^, ^18^, ^19^, ^20^, ^21^, ^22^, ^23^, ^24^, ^25^, ^26^, ^27^ It has also been reported that curcumin has beneficial effects on neurological disorders, neuromuscular diseases, osteoarthritis, and rheumatoid arthritis.^28^, ^29^, ^30^, ^31^ Curcumin can also confer protective effects with regard to exercise‐induced oxidative stress and inflammation, muscle soreness, and muscle recovery and performance in physically‐active individuals.^13^, ^32^, ^33^, ^34^

It has been suggested that most of the beneficial effects of curcumin on different aspects of human health are related to its anti‐inflammatory and anti‐oxidant properties.^13^ Several studies have shown that impairment of nuclear factor erythroid‐2 related‐factor‐2 (Nrf2) expression, which can occur during aging, is associated with an increase in oxidative stress and muscle degeneration.^35^, ^36^ It has also been confirmed that free radicals are directly quenched, or inactivated, by curcumin.^37^ In addition, curcumin has a salient role in the activation of Nrf2.^38^, ^39^ Indeed, curcumin has been demonstrated to increase the expression and activation of Nrf2 *via* dissociation of Nrf2 from kelch‐like ECH‐associated protein 1 (Keap1).^40^ Importantly, up‐regulation Nrf2 in skeletal muscle has been induced in response to curcumin intake.^41^

Curcumin also provides beneficial effects in conditions that can result in skeletal muscle wasting, such as during sepsis and inflammation.^42^, ^43^, ^44^ In addition to the antioxidant activities of curcumin, it has been recently suggested that the beneficial effects of curcumin on gut microbiota might decrease muscle fatigue after exercise. In a recent animal study, it was shown that nano‐curcumin had a favourable effect on muscle fatigue after swimming, which was suggested to occur through alteration of the composition of the gut microbiota, and resulted in an increase in swimming time, forelimb grip strength, and tissue glycogen content.^45^

Another hypothesis regarding the effects of curcumin on skeletal muscle is the ability of curcumin to increase mitochondrial biogenesis.^46^ It was reported that treatment with curcumin, in addition to exercise, resulted in an increase in cAMP levels in skeletal muscle *via* a reduction in the phosphorylation of PDE4A. Curcumin supplementation in conjunction with exercise can increase downstream targets of protein kinase A (PKA), including the phosphorylation of 5′ adenosine monophosphate‐activated protein kinase (AMPK), the deacetylation of peroxisome proliferator‐activated receptor gamma coactivator 1‐alpha (PGC‐1α), and the expression of cytochrome c oxidase‐IV (COX‐IV) in animal skeletal muscle, which suggests that curcumin increases mitochondrial biogenesis in muscle.^46^ However, very few studies have been conducted to date to assess the effects of curcumin in the management of sarcopenia.

In a very recent study in healthy elderly subjects (*n* = 30), supplementation with Cureit (a bioavailable form of curcuminoids‐Cureit‐500 mg capsules) for 3 months resulted in a significant increase in handgrip strength (+1.43% in the intervention group, whereas there was a slight decrease in the control group (*P* < 0.001)), weight‐lifting capacity (+6.08% in the intervention group compared with −4.54% in the control group), and walking distance covered before feeling tired (+5.01% in the intervention group compared with 2.09% in the control group; *P* = 0.09), without any adverse effects. In addition, a significant decrease in the time taken to walk a pre‐determined distance was observed for those subjects that took Cureit supplements (−1.15%) when compared with those subjects that received a placebo (+2.02%). However, Cureit had no effects on inflammatory factors such as CRP and ESR^47^ (*Table* 1). In another study, healthy subjects (age >65 years) with apparent loss of muscle strength and tiredness were randomly administered one of three interventions: (i) standard management (exercise and a balanced diet) (*n* = 33), (ii) standard management + one tablet/day of Meriva® [each tablet containing 1 g Meriva® (commercially available Phytosome® of curcumin)] (*n* = 32), or (iii) standard management + one tablet/day of Meriva® + additional supplementation (vitamin D, 800 IU/day; vitamin C, 500 mg/day; Isoleucin, 3 g/day; Carnitine, 1 g/day) (*n* = 22). After 3 months of the interventions, the results indicated that hand grip strength, weight lifting capacity, time and distance before feeling tired after cycling, walking and climbing stairs, overall general fitness, proteinuria, oxidative stress, scores on the Karnofsky scale, and left ventricular ejection fraction were all improved in both intervention groups that used Meriva®, or Meriva® + additional supplementation, when compared with these same parameters for those that received only standard management (*P* < 0.05 for all comparisons).^48^

**Table 1:** The effect of plant‐derived natural products on sarcopenia according to the clinical trials

Author, year, country	Intervention	Dose per day	Treatment duration	Subjects	Study design	Main outcomes	Adverse effects
Varma *et al*. 2020, India^47^	Cureit (bioavailable form of curcuminoids)	500 mg	3 months	Health >65 years subjects (*n* = 30)	RCT	1. Compared with placebo, in the Cureit group, the handgrip strength and the weight‐lifting capacity increased. 2. A significant decreased was observed in time taken to walk the same distance in response to the Cureit supplements. 3. Cureit had no effects on inflammatory factors such as CRP and ESR.	No adverse effects
Franceschi *et al*. 2016, Italy^48^	(i) Standard management: exercise and balanced diet (ii) Standard management + Meriva® one tablet/day (each tablet containing 1 g Meriva® (commercially available Phytosome® of curcumin)) (iii) Standard management + Meriva® one tablet/day + other supplementation (vitamin D 800 IU/day; vitamin C 500 mg/day; Isoleucin 3 g/day; Carinitine 1 g/day).	1 gram	3 months	Health >65 years subjects with apparent loss of strength and tiredness (*n* = 87)	Clinical trial	Compared with standard treatment, the hand grip, weight lifting, time/distance before feeling tired after cycling, walking and climbing stairs; general fitness, proteinuria, oxidative stress, Karnofsky scale; left ventricular ejection fraction improved in the other two intervention groups (ii and iii) (*P* < 0.05 for all comparisons).	Not reported
Alway *et al*. 2017, USA^49^	Resveratrol	500 mg/day	3 months	Healthy older subjects 65–80 years old (*n* = 30)	Clinical Trial	Resveratrol combined with exercise increased the mitochondrial density, muscle exhaustion endurance, mean fibre area and total myonuclei in comparison with placebo and exercise treatments (*P* < 0.05 for all comparisons).	No adverse effects
Harper *et al*. 2020, USA^50^	Exercise and resveratrol	500 or 1000 mg/day	12 week	Elderly adults with physical function limitations (*n* = 60)	RCT	The 6 min walk test and citrate synthase in the exercise plus 1000 mg resveratrol were higher than other groups.	No adverse effects
Mafi *et al*. 2019, Iran^51^	Resistance training (RT) and epicatechin (EP)	1 mg of pure Epicatechin per 1 kg of their body weight	8 weeks	Males with sarcopenia (*n* = 62)	RCT	There was a significant increase in follistatin, follistatin/myostatin ratio, leg press, and chest press in RT + EP in compared with RT, EP, and PL groups (*P* < 0.05).	No adverse effects
Kim *et al*. 2013, Japanese^52^	(i) Exercise and intake of tea catechin (350 mL/day) (ii) Exercise (iii) Tea catechin supplementation (iv) Health education	350 mL/day	3 months	Elderly women with sarcopenia aged over 75 years (*n* = 128)	RCt	The exercise and catechin group had a meaningful effect in the combined variables of leg muscle mass and general walking activity in comparison with the health education group.	No adverse effects
Regard *et al*. 2020, Japan^53^	(i) Carbohydrate + placebo capsules (CHO) (ii) Whey protein isolate + placebo capsules (iii) Whey protein isolate + bioactives (BIO) capsules containing omega‐3 fatty acids, rutin, and curcumin	Fish oil (1.5 g/day) including 18% eicosapentaenoic acid (EPA) and 7% docosahexaenoic acid DHA and 500 mg/day curcumin	12 weeks	Health >65 years older men and women (*n* = 37)	RCT	There was a significant effect (+13%) on knee expansion force in the whey protein isolate + bioactive group compared with the carbohydrate group (*P* = 0.025). Whey protein isolate alone did not have a significant effect.	Not reported
Orsatti *et al*. 2018, Brazil^54^	(i) Placebo and resistance training (RT) + milk (ii) Soy and RT + milk	25 g soy	16 weeks	Postmenopausal women (*n* = 32)	RCT	A significant improvement in muscle strength, 1RM of bench press, knee extension, total load, and the total load exercises/muscle mass was observed in the combination of soy protein + RT and milk group compared with placebo group (*P* < 0.05).	No adverse effects
Kim *et al*. 2016, Japanese^55^	(i) Nutrition supplement (ii) Nutrition supplement + exercise (iii) Exercise (iv) Health education classes (Control group)	Nutrition supplement contained 3.0 g of leucine supplemented essential amino acid, 20 μg vitamin D, and 540 mg of catechin daily	3 months	Elderly women with sarcopenic obesity (*n* = 139)	RCT	Exercise with nutrition reduced total body fat mass compared with the control group (*P* = 0.036), stride increased in both exercise group compared with nutrition group and vitamin D significantly increased in both nutrition groups compared with exercise and control groups (*P* < 0.001). In addition, walking speed significantly increased in nutrition + exercise groups, and leptin significantly decreased in all intervention groups; however, differences between the study groups were not significant.	No adverse effects
Munguia, *et al*. 2019, Mexico^56^	Cocoa (with or without colouring and flavouring)	179 mg	12 weeks	Subjects aged 55–70 years (n = 60)	RCT	Intake of flavonoids significantly influences blood inflammation, the performance of movement, and quality of life in comparison with group without flavonoids (*P* < 0.05 for all comparisons).	No adverse effects
Park *et al*. 2020, Korea^57^	Korean Red Ginseng	3 mg/kg/day	24 weeks	Type 2 diabetes patients (*n* = 59)	RCT	Korean Red Ginseng had a significant effect on serum sex hormone binding globulin (SHBG) (*P* < 0.001), and follistatin (*P* = 0.004). An attenuated decline in Troponin T (*r* = 0.519, *P* = 0.009) was seen in old postmenopausal women.	No adverse effects

RCT, randomized clinical trial.

In the preclinical area using animal models, prolonged dietary curcumin exposure on muscle mass and function was evaluated in rats. Thirty‐two‐month‐old male F344xBN rats were provided a diet with or without 0.2% curcumin for 4 months. The rats were divided into three groups: (i) control group (*ad libitum*), (ii) 0.2% curcumin, and (iii) pair‐fed rats. The results indicated that, in comparison with the control group, curcumin rats showed lower food intake. These authors used the pair‐fed rats for comparison with rats that ingested the diet containing 0.2% curcumin. Rats that ingested the diet containing 0.2% curcumin displayed larger plantaris mass and force production than rats in the pair‐fed group. These authors also found that nuclear fraction levels of Nrf2 were greater, and oxidative macromolecule damage was lower, in rats that ingested curcumin than in pair‐fed rats. Lastly, there were no significant differences in various measures of antioxidant status between any of the groups at the end of the 4‐month study^58^ (*Table* 2).

**Table 2:** The effect of plant‐derived natural products on sarcopenia according to the pre‐clinical studies

Author, year, country	Intervention	Dose per day	Treatment duration	Animals	Main outcomes
Receno, 2019, USA^58^	(i) Control group (ad libitum) (ii) 0.2% curcumin (iii) Pair‐fed rats	0.2% curcumin	4 months	Rats	1. Curcumin rats had lower food intake compared with control group 2. Compared with paired fed rats, curcumin rats showed more general plantaris mass and strength production 3. A larger nuclear fraction and lower oxidative macromolecule loss were recognized in the curcumin group compared with paired rats 4. Antioxidant status did not change in the study groups
Asami *et al*. 2018, Japan^59^	(i) Normal group (ii) 0.5 resveratrol	0.5% resveratrol diet	3 weeks	Male ICR mice	In mice, resveratrol group significantly inhibits muscle atrophy. It can be because of reducing in atrogin‐1 and p62‐dependent signalling.
Bai *et al*. 2019, Taiwan^60^	(i) High‐fat diet with 50 mg/day resveratrol (ii) High‐fat diet with middle dose of resveratrol (iii) High‐fat diet with high dose of resveratrol	50, 100, and 200 mg/kg BW	2 months	C57BL/6J mice	Mice with a diet with high fat and high dose of resveratrol during sarcopenia with obesity could increase skeletal muscle mass and the expression of mitochondrial capacity connected in Bcl‐2 and PI3K/AKT.
Bai *et al*. 2020, Taiwan^61^	SAMP8 mice were received resveratrol two times a week for 1 month, and exercise training for twice a week	150 mg/kg BW/day	4 weeks	SAMP8 mouse	Resveratrol and its combination with exercise training provoked hypertrophy in skeletal muscles of sarcopenia SAMP8 mice.
Toniolo *et al*. 2018, Italy^62^	Resveratrol	0.04% diet enriched with resveratrol	6 months	C57/BL6 aging mice	The resveratrol‐supplemented diet had less tubular aggregates and much resistance to weakness in an *ex vivo* contraction test.
Zhou *et al*. 2019, China^63^	Resveratrol	150 mg/kg/day	6 weeks	Male SD rats	Only exercise training has an important effect on skeletal muscle capacity and connected expression of gene although, either exercise practice and resveratrol receiving have significant effects on weight loss.
Toniolo *et al*. 2021, Italy^64^	Resveratrol	0.04% RES‐supplemented diet	6 months	C57BL/6J male mice	Treatment with resveratrol increased capillaries per fibre in aging SKM, and it can be thought a good component to prevent capillary rarefaction in tissue related to the SKM.
Ringholm *et al*. 2013, Denmark^65^	Resveratrol	4 g/kg food	12 months	PGC‐1α KO mice	The intervention of resveratrol alone or with training had no significant effect on oxidative stress in skeletal muscle and angiogenic proteins.
Liao *et al*. 2017, China^66^	Resveratrol	150 mg/kg/day	6 weeks	SD rat	There is a significant association between exercise, resveratrol, and their combination and anti‐apoptotic pathways through stimulation of AMPK/Sirt1.
Bennett *et al*. 2013, USA^67^	Resveratrol	(diet including 12.5 mg/kg/day, or oral gavage about 50 mg/kg/day by)	14 days	Male Fisher Norway rats	Resveratrol did not decrease plantaris muscle wet weight through hindlimb suspension and did not interrupt fibre. The resveratrol‐associated rise in the size of type II fibre and muscle mass rehabilitation after interruption.
Pence *et al*. 2016, USA 91	Epigallocatechin‐3‐gallate (EGCG) and ‐alanine (−ALA)	EGCG)1.5 mg/kg) and ALA BALB/cByJ (3.43 mg/kg)	41 days	Mice	Training and supplements differentially regulated expression of the gene in the gastrocnemius of old mice, while running wheel without any dietary supplement increased muscle function. There was no significant effect between EGCG,B ‐alanine, and running wheel.
Meador *et al*. 2015, USA 92	The green tea catechin, epigallocatechin‐3‐gallate (EGCg)	200 mg/kg	8 weeks	Sprague–Dawley rat	Rats with consumption of EGCg maintained muscle in sarcopenic with enhanced anabolic factors expression.
Munguia *et al*. 2020, Mexico^68^	Cocoa drink supplemented with flavanols or pure (−)‐epicatechin	Flavonoids (2 mg EC + 12.8 mg procyanidins/kg bw) or 3) (−)‐epicatechin (2 mg EC/kg bw)	5 weeks	C57BL/6 male mice	The cocoa drink supplemented with flavanols and refined (−)‐epicatechin, enhanced physical appearance, the follistatin/myostatin ratio and enhanced myocyte enhancer factor 2A (MEF2A) expression in compared with the control group.
Hong *et al*. 2020, South Korea^69^	Green tea extracts	1, 5, 10, 15, and 20 μg/mL	24 h	C2C12 mouse myoblast cell	1. These medications significantly improved the genetic expression of myogenin, Myf5, and MyoD. 2. In the intervention group improved the levels of AMP‐activated protein kinase‐α (AMPKα) and muscle RING‐finger protein‐1 (MuRF‐1) in comparison with AICAR and green tea extract groups.
Alwat *et al*. 2015, USA^70^	Green tea extract (GTE)	50 mg/kg BW	35 days	Fischer 344 Brown Norway rats	GTE significantly diminished the loss of hindlimb muscle mass, muscle fibre cross‐sectional area loss in both plantaris, oxidative stress, the profusion of the Bcl‐2‐associated X protein (Bax), and tetanic force in comparison with vehicle treatment through HLS. In contrast, GTE declined improvement of muscle function or mass compared with vehicle treatment.
Cao *et al*. 2007, France^71^	*Ginkgo biloba* extract EGb 761 or Wisconsin Ginseng	100 μg/mL	14 days	Transgenic *C. elegans* strain (PD4251)	Both EGb 761 and Wisconsin Ginseng significantly inhibited sarcopenia.
Christen *et al*. 2007, France^72^	*Ginkgo biloba* EGb 761	75 mg/kg	5 weeks	Wistar rats	The extract of standardized *Ginkgo biloba* EGb 761 led to a decrease of the loss of muscle mass in the older subject.
Bidon *et al*. 2009, France^73^	*Ginkgo biloba* extract		5 weeks	Rats	*Ginkgo biloba* extract increased muscle mass that contributed to the development of the muscular acts.
Chang *et al*. 2019, Japan^74^	Oligonol	200 mg/kg	8 weeks	SAMP8 mice	Oligonol supplementation had a significant reduction in the expression of cytochrome c and cleaved caspase‐9 in skeletal muscle.
Le *et al*. 2014, Korea^75^	Quercetin	0.05 or 0.1 g quercetin for 100 g diet	9 weeks	Male C57BL/6 mice	Quercetin diminishes atrophy in skeletal muscle by repressing receptors of inflammatory and their signalling pathway.

### Resveratrol

Resveratrol is a popular natural compound produced by plants, and first isolated from plant roots used in conventional Chinese and Japanese medicine as an anti‐inflammatory and anti‐platelet agent.^76^, ^77^ This original ‘functional food’ has been identified in more than 70 plant species and is also observed in specific red wines. Resveratrol has significant benefits for human health, such as anti‐cancer, antimicrobial, anti‐aging, anti‐oxidant, anti‐diabetic, and anti‐inflammatory effects.^78^, ^79^, ^80^, ^81^, ^82^, ^83^ Despite some negative findings on the putative cardioprotective actions of resveratrol,^84^, ^85^ some studies have shown that resveratrol, combined with endurance exercise training, can improve the VO_2_ max, as well as fatigue resistance.^49^, ^86^, ^87^ In contrast, other long‐term investigations have reported that resveratrol produced moderate, or no changes, in overcoming diminished muscle mass or muscle inflammation.^49^, ^88^, ^89^ Some clinical trials have shown no, or only insignificant improvements, in overall muscle function.^49^

However, in some experimental studies, resveratrol was demonstrated to enhance muscle protein synthesis,^90^ decrease the degeneration of muscle protein, and attenuate atrophy of skeletal muscle fibres.^91^, ^92^ For example, in rodents administered 400 mg/kg/day resveratrol, it was reported that there was a significant decrease in atrophy of muscle fibre.^93^ However, a low dose of resveratrol (12.5 mg/kg/day) did not induce an increase in the quantity of satellite cells or muscle mass.^67^ Nevertheless, other data that have examined the ingestion of resveratrol in conjunction with exercise has shown that the combination of this natural compound and physical exercise decreases sarcopenia in humans to a greater extent than exercise alone.^94^ Furthermore, it was also reported that resveratrol + exercise enhanced maximal consumption of oxygen and improved mitochondrial volume density more than exercise alone.^94^

Resveratrol treatment has also been shown to not only improve mitochondria volume density but also modulate genes linked with mitochondrial morphology.^49^ In fact, it has been reported that resveratrol influences genes associated with mitochondrial‐regulated apoptotic signalling, which may represent an essential mechanism to exclude dysfunctional organelles.^49^ Resveratrol has a critical role in contractile protein accumulation and muscle strength by improving overall muscle function, as well as causing an enhancement in muscle fibre cross‐sectional area.^49^, ^95^, ^96^ These resveratrol‐mediated changes appear to contribute to the enhancement of satellite cell proliferation in the muscles of old mice.^49^

To assess the effects of resveratrol in sarcopenia, both experimental animal and human studies have been conducted. Beginning with human studies, a randomized controlled trial included 30 healthy adult men and women that received either a placebo, or 500 mg/g resveratrol, for 12 weeks before and after exercise. At the end of the study, resveratrol plus exercise enhanced mitochondrial density, resistance to muscle fatigue, mean muscle fibre area, and total myonuclei when compared with these same indices in the subjects that received the placebo plus exercise (*P* < 0.05 for all comparisons). Additionally, resveratrol treatment led to an improvement in knee extensor muscle peak energy, average peak energy, and muscle strength after exercise, although exercise did not improve these parameters in subjects in the placebo group.^49^

In another recent randomized controlled trial, a total of 60 elderly male individuals with limitations in physical function were assigned to three groups and received either exercise and placebo, exercise with 500 mg/day resveratrol, or exercise and 1000 mg/day resveratrol. After 3 months of intervention, the 6 min walk test, as well as citrate synthase (the activity associated with this enzyme indicates the mitochondrial content of skeletal muscle), were greater in the exercise plus 1000 mg/day resveratrol group when compared with these same parameters in the other two groups (*Table* 1).^50^

As it pertains to experimental animal studies, one animal study utilized male ICR mice, which were randomized into two groups: normal or resveratrol diets for 3 weeks. Results indicated that after denervation, resveratrol significantly lowers the extent of denervation‐induced fibre atrophy and muscle weight. Furthermore, this intervention suppressed denervation‐induced atrogin‐1 and p62 immunoreactivity. In contrast, 0.5% resveratrol did not have any significant effect on total protein amount of atrogin‐1 or p62 in mice with muscle denervation.^59^

In another animal investigation, two mouse models were used. The first mouse model utilized senescence accelerated mouse‐prone 8 (SAMP8) mice, which received resveratrol (150 mg/kg BW daily) two times a week for 1 month, together with exercise training twice a week for the experimental period. The second model used male C57BL/6J mice that were provided a high‐fat diet for 5 weeks to induce obesity and then were randomly assigned to three groups to receive HFD in addition to either a low (50 mg/kg BW), middle (100 mg/kg BW), or high (200 mg/kg BW) dose of resveratrol for 4 weeks. Skeletal muscle atrophy was induced by a HFD, although mice with HFD‐induced obesity that received high‐dose resveratrol showed preservation of mitochondrial activity in skeletal muscle. Compared with control or HFD‐fed mice, high‐dose resveratrol dramatically up‐regulated Bcl 2 and down‐regulated Bad protein expression. Resveratrol attenuated age‐related loss of skeletal muscle mass and mitochondrial function. However, exercise training had no significant effects on sarcopenia in SAMP8 mice.^60^

In a different experimental study by Toniolo *et al*., aged C57/BL6 mice ingested a diet enriched with 0.04% resveratrol for 6 months, while mice that received no treatment were considered the control group. Results showed that mice that ingested the resveratrol‐supplemented diet had less tubular aggregates (TAs), which are a morphological feature with increases in densely‐arranged tubules from the sarcoplasmic reticulum of striated muscles. Resveratrol treatment increased MyHCIIB (myosin heavy chains IIa and IId) expression and enhanced fatigue resistance.^62^ In yet another study, Zhou *et al*. used male Sprague–Dawley rats, which were randomly assigned to three groups and evaluated after 6 weeks. The three groups of rats were as follows: (i) control group, (ii) regular exercise training group, and (iii) a resveratrol‐treated (150 mg/kg/day) group. At post‐intervention, there was no significant difference in the gastrocnemius muscle index and absolute grip strength between the three groups. Furthermore, 12 differentially expressed genes were identified in the resveratrol group compared with the control group. Only exercise training had an important effect on skeletal muscle function and associated gene expression, although both exercise training (group ii) and resveratrol‐treated (group iii) rats exhibited significant weight loss.^63^ In a more recent study by Toniolo *et al*., dietary supplementation with 0.04% resveratrol for 6 months resulted in improved resistance to muscle fatigue. The treatment with resveratrol improved the capillarization of skeletal muscle in aging mice and was suggested to be beneficial in preventing capillary rarefaction in skeletal muscle tissue.^64^

Numerous other studies evaluating the effects of resveratrol and muscle status have used rats as an animal model. In a study by Liao *et al*., the effects of exercise, resveratrol, and exercise + resveratrol was evaluated using aged Sprague–Dawley rats (238). The rats were randomly assigned to three groups. Group 1 received short‐term exercise, while Group 2 was given dietary resveratrol (150 mg/kg/day), and Group 3 received both exercise training and dietary resveratrol (150 mg/kg/day) for a duration of 6 weeks. After 6 weeks of intervention, these authors showed that exercise, resveratrol, or the combination of both exercise and resveratrol significantly increased the relative grip strength and gastrocnemius muscle mass in the aged rats. Although electron microscopy showed that the aged rats had a significant increase in sarcomere length, I‐band, and H‐zone, the interventions of exercise, resveratrol, or their combination significantly reduced the increase observed in these three parameters. Additionally, although light microscopy revealed a significant increase in Feret's diameter (a measurement used to quantify the size of cells in tissue sections), as well as the muscle cell's cross‐sectional area in aged rats, exercise and resveratrol did not show a significant effect on either parameter. Lastly, this study by Liao *et al*. showed that exercise, resveratrol, or exercise + resveratrol significantly increased the expression of p‐AMPK and SIRT1 and decreased the expression of acetyl P53 and the Bax/Bcl‐2 ratio in the aged rats. These authors concluded that aged rats exhibit significant changes in gastrocnemius muscle morphology and ultrastructure and that the protective effects of exercise, resveratrol and their combination are probably associated with anti‐apoptotic signalling pathways *via* activation of AMPK/Sirt1.^66^

Finally, a study using 36 male Fisher rats (32 months old) were treated with either a water vehicle or 50 mg/kg/day resveratrol *via* oral gavage. A total of six rats from the vehicle and the resveratrol‐treated groups were used to evaluate hindlimb suspension and recovery. Additionally, the control animals for the hindlimb suspension and reloading groups consisted of two groups of six vehicle‐treated animals that maintained normal ambulation throughout the experiment. The findings in this study demonstrated that resveratrol supplementation was unable to attenuate the decreases in plantaris muscle wet weight during hindlimb suspension, although it did improve muscle mass during reloading following hindlimb suspension. While resveratrol did not prevent fibre atrophy during the period of disuse, it increased the fibre cross‐sectional area of type IIA and IIB fibres in response to reloading after hindlimb suspension. These authors concluded that resveratrol appeared to have a modest therapeutic benefit for improving muscle mass after muscle disuse in aged rats^67^ (*Table* 2).

### Green tea

Green tea is obtained from the leaves of *Camellia sinensis* ^97^ and is a traditional drink used for its beneficial effects in cardiovascular diseases^98^ and various other chronic diseases.^98^, ^99^, ^100^ This nutraceutical is chemically classified as a polyphenol and contains many catechins, such as [epicatechin (EC), epigallocatechin (EGC), epicatechingallate (ECG), and epigallocatechin gallate (EGCG)]. These compounds exert anti‐inflammatory effects by reducing the expression of nuclear factor‐κB and the production of proinflammatory cytokines.^98^ In addition, green tea extract (GTE) has been reported to counter insulin resistance and hypertension *via* its antioxidant and anti‐inflammatory properties.^101^, ^102^ It has been suggested that two fragrant ring structures that are joined by an oxygenated heterocycle containing a 4‐hydroxyl group is the chemical moiety in GTE that influences several different signalling pathways.^69^, ^103^ Some previous investigations have shown that GTE improves overall muscle function.^69^ Baik *et al*. demonstrated that GTE significantly improved the biotransformation of catechins, and that pectinase‐driven hydrolysis significantly enhanced interleukin‐6 (IL‐6) production in macrophages.^104^ It is thought that the functional (biologically‐active) compounds in GTE are related to the control of systemic inflammation and may potentially reduce the symptoms of muscle dysfunction.^105^

One study reported the effect of green tea on the protein levels of MHC, MyoD, and myogenin, as well as the activation of promyogenic signalling pathways, p38 MAPK and Akt, in C2C12 myoblasts treated with (−)‐epicatechin (EC).^106^ The findings in this study suggested that EC treatment promotes myogenic differentiation by activation of key promyogenic signalling pathways and MyoD‐mediated gene expression. It may have potential for the treatment of muscle weakness and muscle atrophy. Gutierrz‐Salmean *et al*. established that intervention with green tea resulted in a significant improvement in the levels of MEF2, Myf5, MyoD, and myogenin in the skeletal muscles of old EC‐treated mice (25 months), as well as increased muscle strength in human hands.^69^

Turning to the effects of green tea in humans, in a recent double‐blind controlled study, 62 males were randomly assigned to four groups to receive either resistance training (RT), epicatechin (EC) in a dose of 1 mg of pure epicatechin per 1 kg of their body weight, resistance training + epicatechin (RT + EC), or placebo (PL) for 8 weeks. Results showed that there was a significant increase in follistatin, the follistatin/myostatin ratio, leg press muscle strength, and chest press muscle strength in the subjects contained in the RT + EC group when compared with these same measurements in subjects contained in the RT, EC, and PL groups (*P* < 0.05)^51^ (*Table* 1). In a randomized controlled trial, 128 women with sarcopenia were randomized into four groups: (i) exercise and intake of tea catechin (350 mL/day), (ii) exercise, (iii) tea catechin supplementation, and (iv) health education for 3 months. At post‐intervention, the exercise and catechin group had a significant effect in the combined variables of leg muscle mass and usual walking speed in comparison with the health education group.^52^

Although human clinical trials provide promising results of tea catechins and overall muscle strength and performance, studies using experimental animal models typically afford a greater understanding of the molecular mechanisms involved with a chemical agent derived from, or contained in, a natural product and its effects on muscle function. For example, in a study using aged BALB/cByJ mice, the animals received either AIN‐93M (standard feed/chow), or AIN‐93M containing 1.5 mg/kg epigallocatechin‐3‐gallate EGCG and 3.43 mg/kg B‐alanine, with or without time on a running wheel, for 41 days. Analysis of the results following the interventions showed that physical training on the running wheel and the supplements appeared to differentially regulate gene expression in the gastrocnemius muscle of aged mice, while physical exercise on the running wheel without any dietary supplements increased muscle function. There was no synergistic effect between the EGCG + B‐alanine diet and physical exercise on the running wheel.^107^ In another study using a rat model of sarcopenia, Sprague–Dawley rats were allowed to consume either a control diet, or a diet fortified with 200 mg/kg of body weight of the green tea catechin, epigallocatechin‐3‐gallate (EGCg), for 2 months. After the intervention, there was a significant increase in gastrocnemius muscle mass, muscle fibre cross‐sectional areas, and muscle mRNA expression of IL‐15 and IGF‐1 with the aged rats that consumed the diet enriched with EGCg when compared with these same parameters in aged rats that consumed the control diet. In comparison with younger adult rats (6 month), the protein expression of 19S, 20S, MuRF1, MAFbx, and myostatin were increased between four‐fold and 12‐fold in the aged controls, but only up to approximately two‐fold in the aged rats that received the diet containing EGCg.^108^ These authors concluded that dietary EGCg supplementation was able to preserve muscle in aged sarcopenic rats by both an attenuation of protein degradation *via* the ubiquitin‐proteasome pathway, together with increased expression of anabolic factors.

In another recent study, the effects of tannase‐converted green tea extract in five doses (1, 5, 10, 15, and 20 μg/mL), or green tea extract (1, 5, 10, 15, and 20 μg/mL), on myotube density and fusion in normal and oxidative stress‐induced C2C12 skeletal muscle cells was evaluated and compared with the results for the AMPK activator 5‐aminoimidazole‐4‐carboxamide‐1‐β‐d‐ribonucleoside (AICAR). The results demonstrated that the myotube density of normal and oxidative stress‐induced C2C12 cells was significantly greater in the tannase‐converted green tea extract‐treated group than that observed in the other groups. Additionally, the tannase‐converted green tea extract and green tea extract treatments significantly up‐regulated the genetic expression of myogenin, Myf5, and MyoD. Lastly, these authors found that the levels of AMPKα and muscle RING‐finger protein‐1 (MuRF‐1) in the tannase‐converted green tea extract group were higher than those in the AICAR and green tea extract groups. Based on these findings, the authors concluded that tannase‐converted green tea extract can be used in the treatment of sarcopenia.^69^

Individuals that do not regularly use their muscles with aging often experience challenges to the recovery in the function of those muscles. This section of the review has been dedicated to the effects of GTE on overall muscle health, and it is important to point out that GTE does not always provide beneficial effects as it pertains to the recovery of sedentary muscles in older adults with sarcopenia. Alway *et al*. studied the effects of GTE on muscle recovery following disuse in aged (32 months) Fischer 344 Brown Norway rats. The rats received either 14 days of hindlimb suspension (HLS), or 14 days of HLS followed by normal ambulatory function for 14 days (i.e. recovery/reloading), while additional animals served as cage controls. The rats were administered either GTE (50 mg/kg body wt), or water (vehicle), by oral gavage 7 days before and throughout the experimental periods. Compared with vehicle treatment, GTE significantly attenuated the loss of hindlimb plantaris muscle mass and tetanic force during HLS. Even though rats that received GTE failed to further improve recovery of muscle function or mass compared with vehicle treatment, they did maintain a smaller loss of muscle mass. Additionally, compared with vehicle treatment, GTE attenuated muscle fibre cross‐sectional area loss in both plantaris and soleus muscles after HLS. GTE also increased satellite cell proliferation and differentiation (critical for muscle repair to occur) in plantaris and soleus muscles during recovery (reloading) from HLS compared with vehicle‐treated muscles, yet this did not further improve muscle recovery. These authors concluded that green tea‐induced changes in the number of satellite cells is, by itself, insufficient to improve muscle recovery following a period of atrophy in old rats^70^ (*Table* 2).

### *Ginkgo biloba* extract

*Ginkgo biloba* extract (GBE) contains terpenoids, flavonoids, alkylphenols, polyphenols, and organic acids,^109^ and is generally used for the prevention and management of cardiovascular disease, neurological disorders (e.g. Alzheimer's disease), ischaemic conditions (e.g. cerebral stroke), and peripheral vascular disease. The use of GBE for the aforementioned conditions is due to the fact that it exhibits both antioxidant and antiplatelet properties.^110^ Experimental and clinical investigations have confirmed the beneficial effects of GBE on glucose and lipid metabolism. In one study, it was reported that sarcopenic rats were treated with EGb 761 (an extract derived from *G. biloba* ), which had a protective effect on sarcopenia by increasing the isometric contractile strength of soleus muscle.^73^ Using DNA microarray analysis, this investigation also showed that EGb 761 mediated transcriptional effects on genes relevant to the regeneration of muscle, as well as energy production by muscles.^73^ Because EGb 761 confers beneficial effects on mammalian muscle, it has been proposed that it could potentially be useful for the management of sarcopenia.^73^, ^111^

In another study, a transgenic *C. elegans* nematode (strain PD4251) was fed either EGb 761 (100 μg/mL), or Wisconsin Ginseng (100 μg/mL), and muscle integrity of body wall muscles was assessed. This particular experimental model, which uses *C. elegans* worms, was created to study age‐associated deterioration of body wall muscle cells. After 14 days of feeding either EGb 761, or Wisconsin Ginseng to the nematodes, results showed that both EGb 761 and Wisconsin Ginseng significantly delayed the development of sarcopenia. Additionally, both agents ameliorated the age‐associated decline of locomotive behaviours including locomotion, body bend, and pharyngeal pumping, although Ginseng was more effective in worms of more advanced age.^71^

In a different study using rats, young (4 months) and older (22 months) Wistar rats were randomly divided into three groups (Group 1 = young rats; Groups 2 & 3 = older rats). The young rats received no intervention, whereas the two groups of older rats received either normal drinking water, or drinking water containing 75 mg/kg of body weight of an extract of *Ginkgo biloba* (EGb 761) for 5 weeks. At the end of the study, the older rats that ingested drinking water containing EGb 761 (Group 3) demonstrated a significant decrease in the loss of soleus muscle mass when individually compared with soleus muscle mass for rats in groups 1 and 2.^72^ In a final study, GBE was shown to directly increase soleus muscle mass in rats, which contributed to greater strength and performance of the muscle as evaluated using biochemical and electrophysiological tests^73^ (*Table* 2).

### Other nutraceuticals

Herbal compounds (curcumin and rutin), antioxidants (vitamin E, vitamin A, zinc, and selenium), and protein supplementation have important effects on muscle protein structure in rats.^53^ There are a few clinical studies in humans that have shown that omega‐3 polyunsaturated fatty acids (ω3‐PUFAs) can enhance muscle mass and functionality.^112^, ^113^, ^114^ In addition, the isoflavones in soy protein are of therapeutic benefit for several health disorders associated with menopause, such as inflammation,^54^, ^115^ loss of bone mass,^115^, ^116^ and vasomotor symptoms.^117^ In a recent double‐blind, randomized, controlled study, 37 elderly individuals were recruited into three groups and orally ingested either (i) carbohydrate + placebo capsules (*n* = 12); (ii) whey protein isolate + placebo capsules (*n* = 15); or (iii) whey protein isolate + bioactive (BIO) capsules containing ω3‐PUFAs, rutin, and curcumin (*n* = 10) for 12 weeks. After the intervention, there was a significant increase in knee extension strength in the whey protein isolate + bioactive group (+13%) compared with the carbohydrate + placebo group (+5%) (*P* = 0.025). However, whey protein isolate alone did not have a significant effect in terms of increasing knee extension strength.^53^

In a different double‐blind, randomized, controlled study, 32 postmenopausal women were randomly assigned to two groups and asked to consume either 25 g soy protein in milk + resistance training (*n* = 16), or 25 g of maltodextrin (placebo) in milk + resistance training (*n* = 16), for 16 weeks. At post‐intervention, a significant improvement in muscle strength, 1RM of bench press, knee extension, total load, and the total load exercises/muscle mass was observed in the group that consumed the soy protein and engaged in resistance training (*P* < 0.05).^54^ In yet another randomized controlled trial, 139 obese sarcopenic women aged >70 years were assigned to receive either exercise alone, nutritional supplementation alone (3.0 g of leucine, 20 μg vitamin D, and 540 mg of catechin daily), exercise + nutritional supplementation, or health education only (control group), for 3 months. Results showed that exercise with nutrition reduced total body fat mass compared with the control group (*P* = 0.036), stride increased in both exercise group compared with nutrition group, and vitamin D significantly increased in both nutrition groups compared with exercise and control groups (*P* < 0.001). In addition, walking speed significantly increased in nutrition + exercise groups, and leptin significantly decreased in all intervention groups; however, differences between the study groups were not significant^55^ (*Table* 1).

Another natural product, namely, ginseng ( *Panax ginseng* C.A. Meyer), has been extensively used as an herbal therapeutic compound in East Asia. Specifically, this compound has beneficial effects with regard to blood sugar, oxidative stress, blood lipid profiles, aging, cancer, and postmenopausal health problems in diabetic and non‐diabetic subjects.^57^, ^118^, ^119^, ^120^, ^121^ However, the influence of ginseng on sarcopenia is not yet clear. In a recent randomized, controlled trial, 59 diabetic patients were assigned to receive either Korean Red Ginseng (3 g/kg/day), or placebo, for 24 weeks. At the end of the study, there was a significant improvement in serum sex hormone binding globulin (SHBG) (*P* < 0.001) and follistatin (*P* = 0.004). Subgroup analysis in >55 years postmenopausal women showed an improvement in SHBG, follistatin, growth differentiation factor 15 (GDF‐15), as well as an attenuated reduction in troponin‐T, in response to consumption of Korean Red Ginseng^57^ (*Table* 1).

Oligonol, derived from lychee, contains catechins, procyanidins, and other phenolic compounds and has been demonstrated to decrease diabetes‐associated obesity.^122^ In a previous study, oligonol was shown to decrease muscle loss in mice with diabetes and obesity by suppressing Atrogin‐1 and MuRF1.^123^ In a study conducted by Chang *et al*., it was determined that most pharmacological effects associated with oligonol were shown to be related to restoration of mitochondrial DNA copy number, as well as genes related to mitochondrial biogenesis, which consist of nuclear respiratory factor 1 (Nrf1) and mitochondrial transcription factor A (Tfam).^74^ These authors used SAMP8 mice, which were randomly divided into two groups, and received either a chow diet, or a chow diet containing oligonol (200 mg/kg), for 2 months. At post‐intervention, oligonol enhanced phosphorylation of AKT/mTOR/p70sk6, reduced transcription of muscle RING‐finger protein‐1 (MuRF‐1) and muscle atrophy F‐box (MAFbx) in skeletal muscle, and nuclear localization of Forkhead box O3a (FoxO3a) and NFκB. Additionally, the abundant accumulation of autophagosomes and lysosomes in the skeletal muscle of SAMP8 mice was limited by oligonol. Lastly, oligonol supplementation caused a reduction in the expression of released cytochrome c and cleaved caspase‐9 in the skeletal muscle of SAMP8 mice^74^ (*Table* 2).

Another nutraceutical that has relevance to muscle function is the flavonoids found in cocoa. Some studies have shown that consumption of cocoa flavonoids has positive effects with regard to dyslipidaemia, insulin resistance, and inflammation.^124^ Cocoa flavonoids have been shown to improve the physical functional performance of inactive adults, while also stimulating upstream regulators of mitochondrial structure and function and thereby reducing oxidative stress.^56^ In a double‐blind, randomized, controlled study, 60 individuals were randomly assigned to three groups and asked to consume the following beverages once/day: (i) placebo containing a cocoa‐free skim milk‐based powder with flavouring; (ii) a natural cocoa powder without flavonoids; and (iii) a natural cocoa powder with a high content of flavonoids (about 179 mg). After 12 weeks of intervention, a comprehensive (aging relevant) set of end points were assessed, which included the mean change in indicators of plasma metabolic and oxidative stress, physical performance tests, and overall quality of life^56^ (*Table* 1). The results showed that the regular consumption of flavonoids positively affects plasma oxidative stress and inflammation end points, cardiometabolic risk markers, physical performance, and quality of life (*P* < 0.05 for all comparisons).

On the other hand, literature regarding the use of traditional Chinese medicine reported that Kampo medicine may help to mitigate the extent of frailty development in elderly individuals. In this regard, Go‐sha‐jinki‐Gan (GJG) showed an anti‐sarcopenic effect in SAMP8 mice *via* the insulin/IGF‐1 signalling pathway, which included an improvement in mitochondrial function and a decrease in the production of TNF‐alpha.^125^ In addition, it was reported that GJG improved the neuropathic pain associated with allodynia in a chronic constriction injury mouse model by suppressing the expression of TNF‐alpha derived from activated microglia.^126^

**Figure 3:**
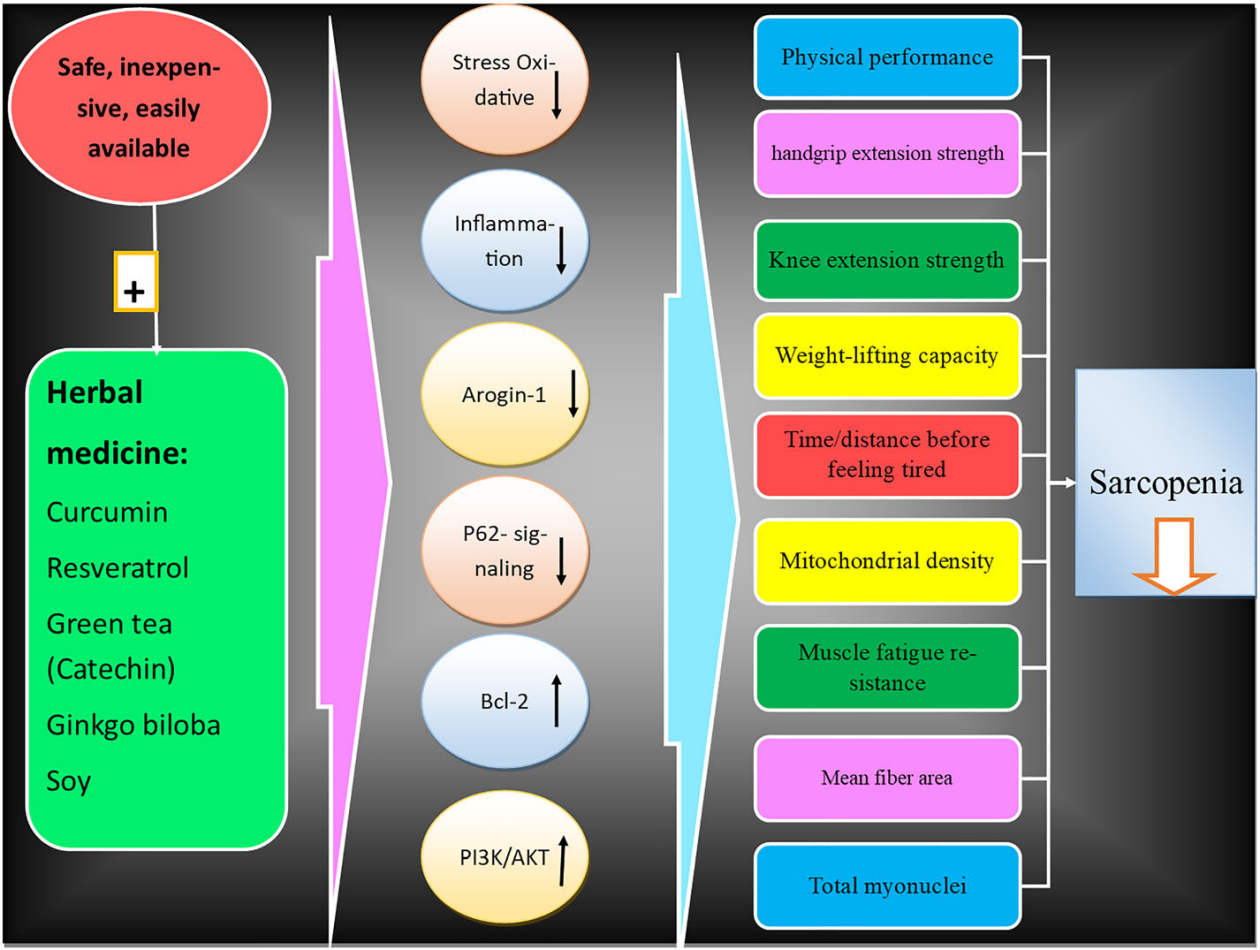
Schematic summary of pathways depicting the possible effects of herbal medicine on sarcopenia and its potential related mechanisms. Bcl‐2, B‐cell lymphoma 2; PI3K, phosphatidylinositol 3‐kinase; Akt, protein kinase B.

Lastly, quercetin (3,3′,4′,5,7‐pentahydroxyflavone) is another nutraceutical and is considered a flavonoid compound. It has been reported that this compound also imparts various beneficial effects on inflammation and oxidative stress.^127^ For example, quercetin decreases inflammation of adipose tissue by inhibiting macrophage growth and cytokine release associated with obesity,^75^, ^128^ as well as inhibits oxidative stress‐induced skeletal muscle atrophy *via* its action on skeletal muscle cells.^129^ In a previous study, male C57BL/6 mice were randomly divided into four groups and received either a regular diet, a high‐fat diet (HFD), a HFD enriched with 0.05% quercetin, or a HFD enriched with 0.1% quercetin. After 9 weeks of intervention, those mice that ingested a HFD with quercetin (mice were obese after 9 weeks of the HFD) had a significant decrease in the levels of inflammatory cytokines, macrophage accumulation, and levels of inflammatory receptors in skeletal muscle. Supplementation with quercetin also reduced transcript and protein levels of specific atrophic factors, Atrogin‐1 and MuRF1, in the skeletal muscle of the HFD‐fed obese mice, as well as protected against the reduction of muscle mass and muscle fibre size. Results of *in vitro* experiments using cocultured myotubes/macrophages in the aforementioned study provided supportive evidence that quercetin markedly diminished transcript levels of inflammatory receptors and activation of their signalling molecules (ERK, p38 MAPK, and NF‐κB), and this was accompanied by a reduction in the expression of the atrophic factors. These authors concluded that the *in vivo* and *in vitro* findings support the hypothesis that quercetin reduces obesity‐induced skeletal muscle atrophy by inhibiting inflammatory receptors and their signalling pathway. Quercetin may be potentially helpful in the prevention of obesity‐induced muscle inflammation and sarcopenia^75^ (*Table* 2) (Figure 3).

## Conclusion

Age‐related sarcopenia is a physical disorder that causes significant mortality and one of the most important public health problems among the elderly community. However, one of the greatest challenges is the diversity in cutoff values for defining sarcopenia. Numerous factors have been proposed to play a role in the pathophysiology of sarcopenia, which include genetic aspects, chronic inflammation, hormone imbalance, and suboptimal nutritional status. There is an urgent need for comprehensive studies in humans to determine the effect of these various factors and the relationship between them.

Concerning the treatment of sarcopenia, there is clinical evidence supporting the substantial beneficial effects of some plant‐derived natural products on sarcopenia, including curcumin, resveratrol, catechins, soy protein, and ginseng. Herbal agents have been shown to impart beneficial effects on various indices used to characterize sarcopenia, which include overall physical performance, handgrip and knee extension strength, weight‐lifting capacity, time/distance feeling tired, resistance to muscle fatigue, mitochondrial density, mean muscle fibre area, and total myonuclei. Importantly, several studies have shown that oxidative stress, inflammation, and quality of life were all improved in response to the regular ingestion of herbal bioactive compounds.

In animal studies, consumption of herbal compounds has been shown to be associated with increased plantaris muscle mass, resistance to fatigue, and increased production of muscle force/strength, as well as inhibition of muscle atrophy. Several molecular mechanisms have been demonstrated in these preclinical studies, such as a reduction in atrogin‐1 and p62‐dependent signalling and increased mitochondrial function in relation to Bcl‐2 and PI3K/AKT (Figure 3). Another molecular mechanism that has been determined from preclinical research on sarcopenia is the favourable effects of herbal products on anti‐apoptotic signalling pathways *via* the activation of AMPK/Sirt1, as well as an increase in the expression of anabolic factors.

Altogether, this review highlights the beneficial effects of medicinal plants/herbal natural products in the treatment of sarcopenia without any adverse side effects of these agents. Nevertheless, due to limited human trials, the clinical benefits of herbal medicine discussed in this review and elsewhere remain inconclusive and cause us to suggest that comprehensive longitudinal studies need to be conducted to better understand the interaction of the various risk factors for sarcopenia that have been presented in this review. Additionally, well‐designed clinical trials are needed to determine therapeutic interventions for sarcopenia that are based on a thorough comprehension of its underlying pathophysiology.

## Conflict of interest

None declared.
